# Supplementary material for: Tastes and retronasal odours evoke a shared flavour-specific neural code in the human insula
Source: Nat Commun. 2025 Sep 12;16:8252. doi: 10.1038/s41467-025-63803-6 (PMC12432251; doi:10.1038/s41467-025-63803-6)
Supplement: Supplementary file 1 — Supplementary Information [file 41467_2025_63803_MOESM1_ESM.pdf]

Tastes and retronasal odours evoke a shared flavour-specific neural code in the human insula

Supplementary Information

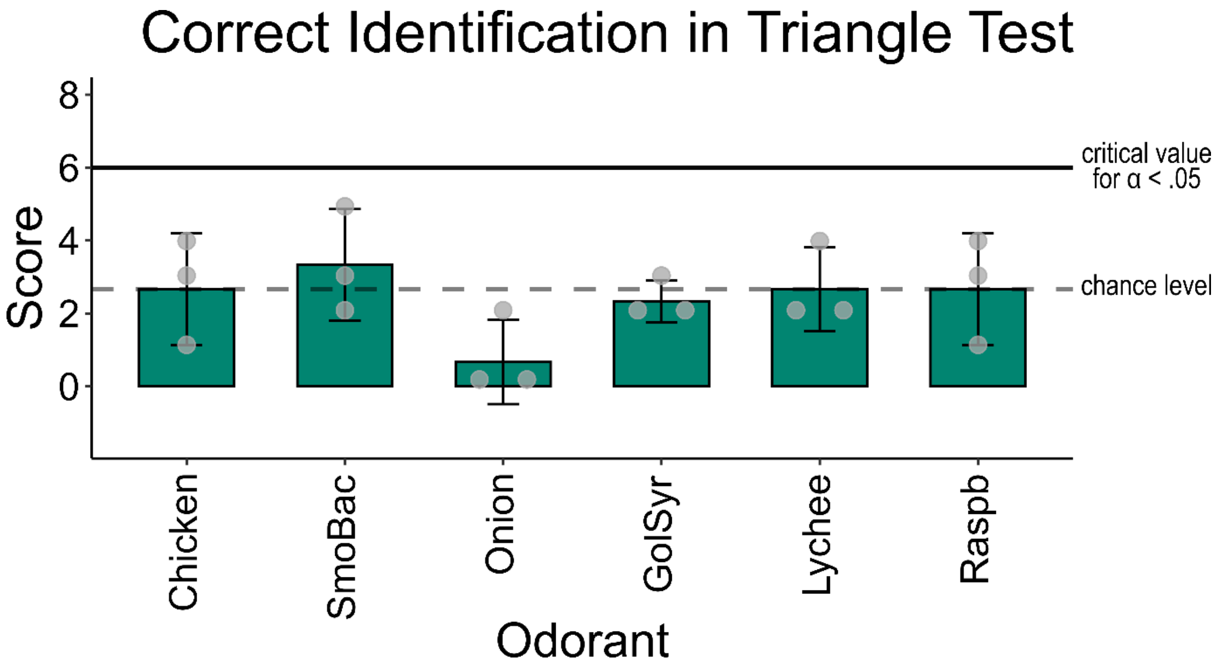

Supplementary Figure 1. Nose-clipped volunteers could not reliably distinguish odorant-containing solutions during triangle testing of aqueous odorants. Grey dots indicate scores of individual volunteers; height of bars indicate mean correct guesses; error bars indicate standard deviation of correct guesses; dashed line indicates chance level (2.67); solid black horizontal line indicates critical threshold for one-tailed binomial test at α < .05 and chance level of 0.33; SmoBac – Smoky Bacon; GolSyr – Golden Syrup; Rasp – Raspberry. N = 3 independent volunteers for each odorant.

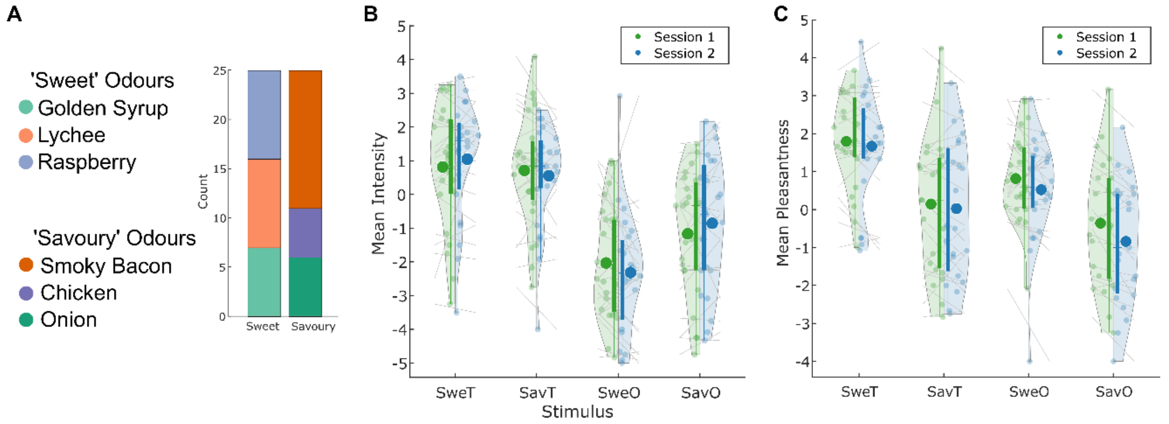

Supplementary Figure 2. Distribution of odours and behavioural ratings. **A)** Distribution of the number of participants assigned to the various 'sweet' and 'savoury' odours showing roughly equal split among 'sweet' odours. For the majority of participants, Smoky Bacon was used as the 'savoury' odour. N = 25 participants. **B)** Group-level and participant-level intensity ratings by session. N = 23 participants. **C)** Group-level and participant-level pleasantness ratings by session. N = 23 participants. While individual ratings changed, mean ratings remained largely consistent at the group-level for both pleasantness and intensity. Large dots signify means; small dots signify individual means; box limits indicate the range of the inner 50% of the data; whiskers signify range; grey lines indicate subject-level changes across sessions. SweT – sweet taste; SweO – sweet odour; SavT – savoury taste; SavO – savoury odour.

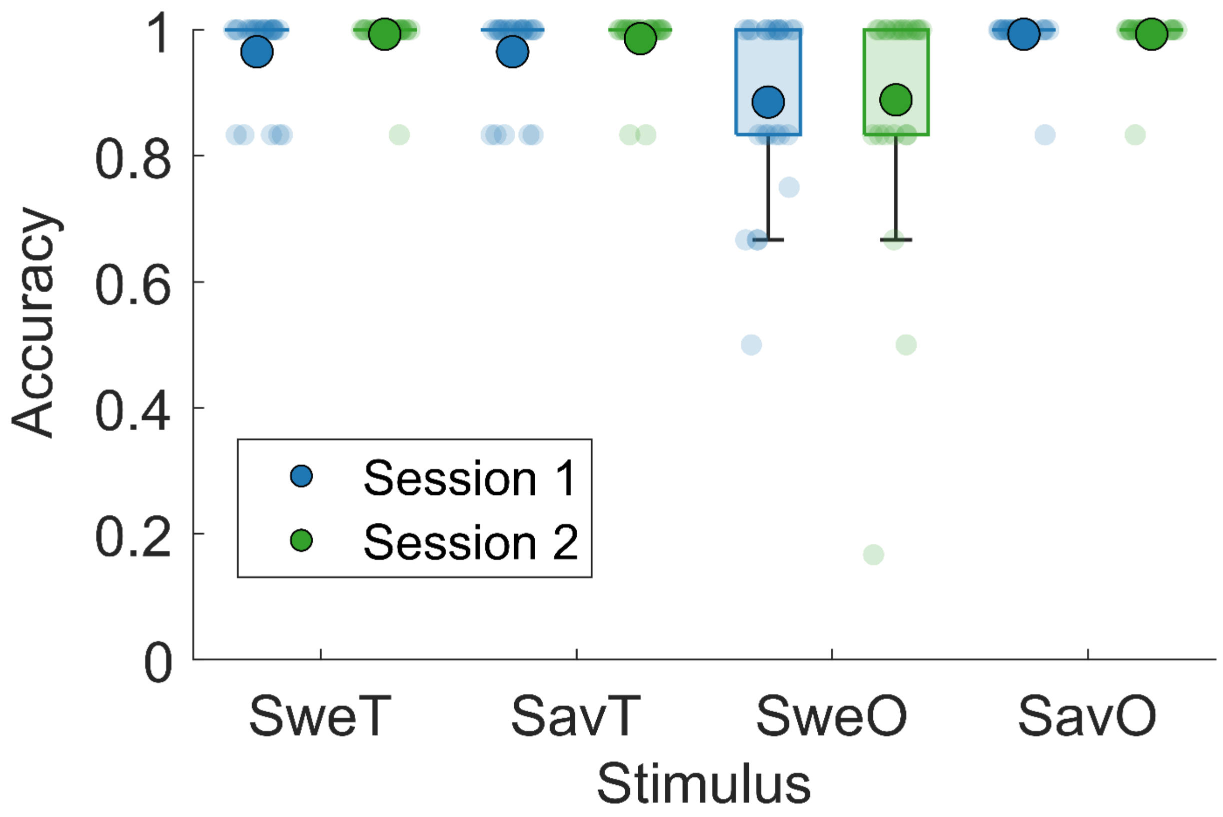

Supplementary Figure 3. Mean identification accuracy of stimuli by session and stimulus. Middle lines on box plots signify median; boxes signify interquartile range; whiskers signify range (1.5 × IQR); translucent scatter signifies individual subject performance; solid dots signify means. N = 23 participants. SweT – sweet taste; SweO – sweet odour; SavT – savoury taste; SavO – savoury odour.

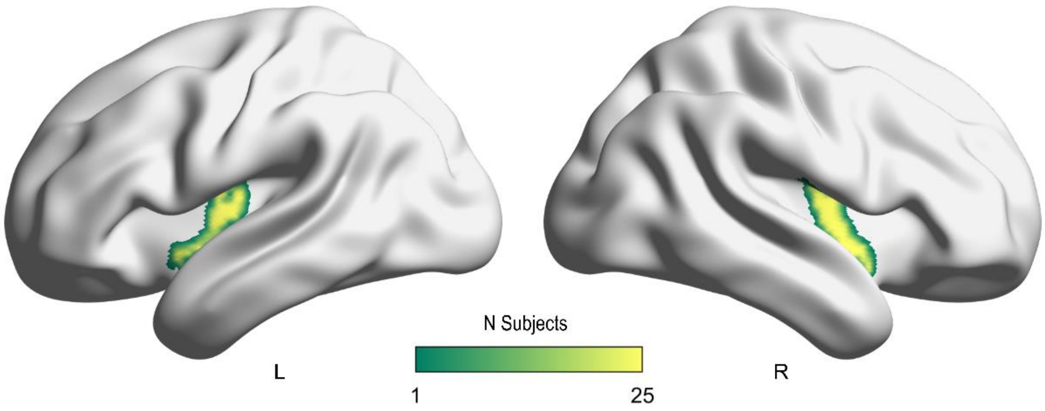

Supplementary Figure 4. Render of cortical surface and heatmap showing the number of subjects contributing to specific sections of the insular cortex. Brighter colours indicate more contributing subjects. This between-subject variability contributed to variability in the leave-one-subject-out ROI, with some subjects extending further posterior and dorsally and others more ventral and anterior. N = 25 participants.

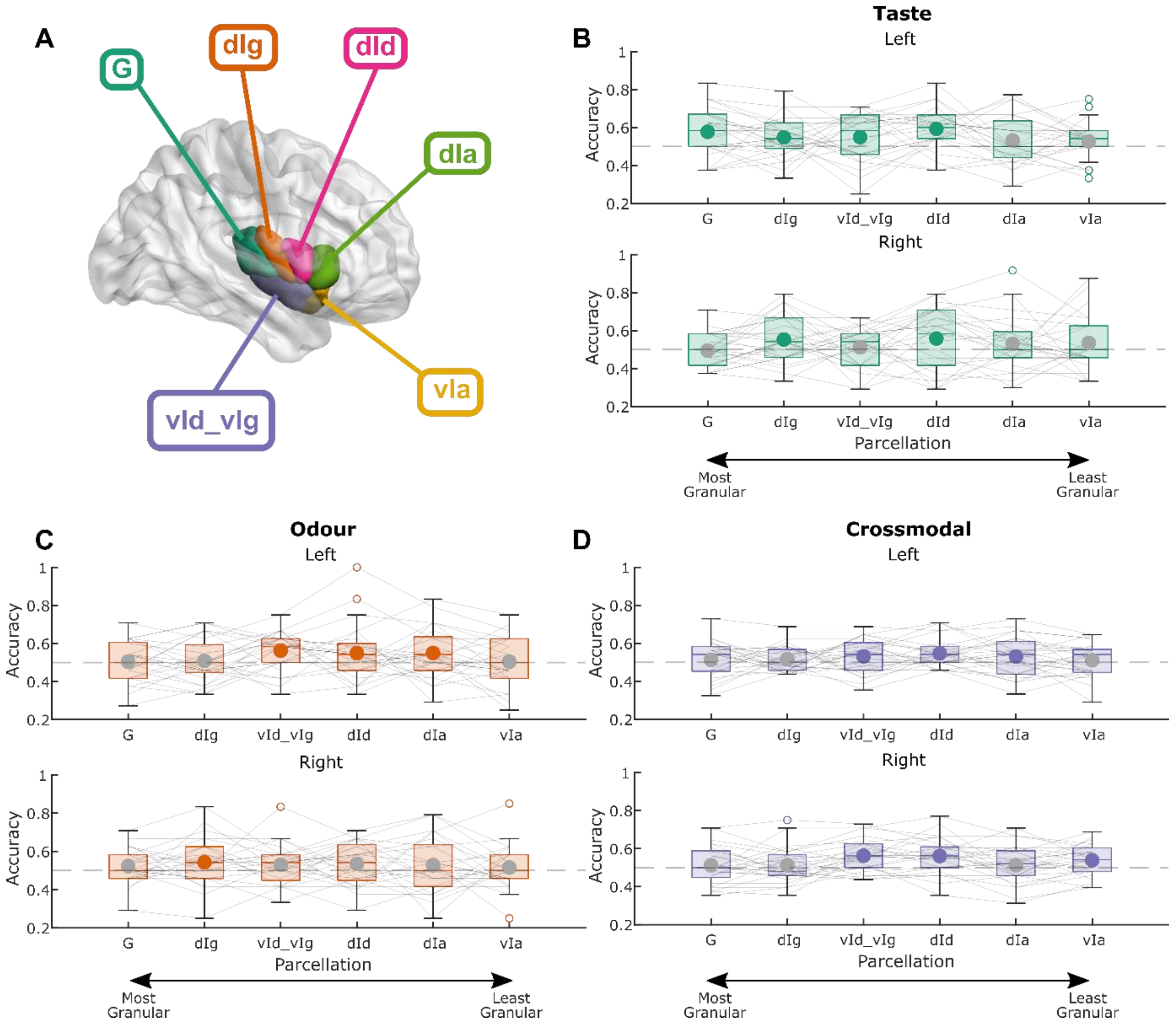

Supplementary Figure 5. Decoder performance separates by partitioning, laterality and parcellations in the Brainnetome Atlas. **A)** Visualisation of each parcellation in the Brainnetome Atlas in one hemisphere. **B)** Mean performance of the tastant decoder tends to be higher in more granular regions. **C)** Mean performance of the odorant decoder tends to be higher in the dysgranular and agranular areas **D)** Mean performance of the crossmodal decoder tends to be stronger than the odorant decoder and occupies similar areas. Middle lines on box plots signify median; boxes signify interquartile range; whiskers signify range (1.5 × IQR); grey lines link individual point; solid dots signify means; coloured mean dots are significant at uncorrected *p* <.05 (uncorrected one-tailed comparisons to permuted null distribution); grey mean dots are not significantly different from the null distribution. N = 25 participants. Precise *p*-values can be found in the Source Data file attached. G – hypergranular insula; dIg – dorsal granular insula; vId_vIG – ventral granular and dysgranular insula; dId – dorsal dysgranular insula; dIa – dorsal agranular insula; vIa – ventral agranular insula.

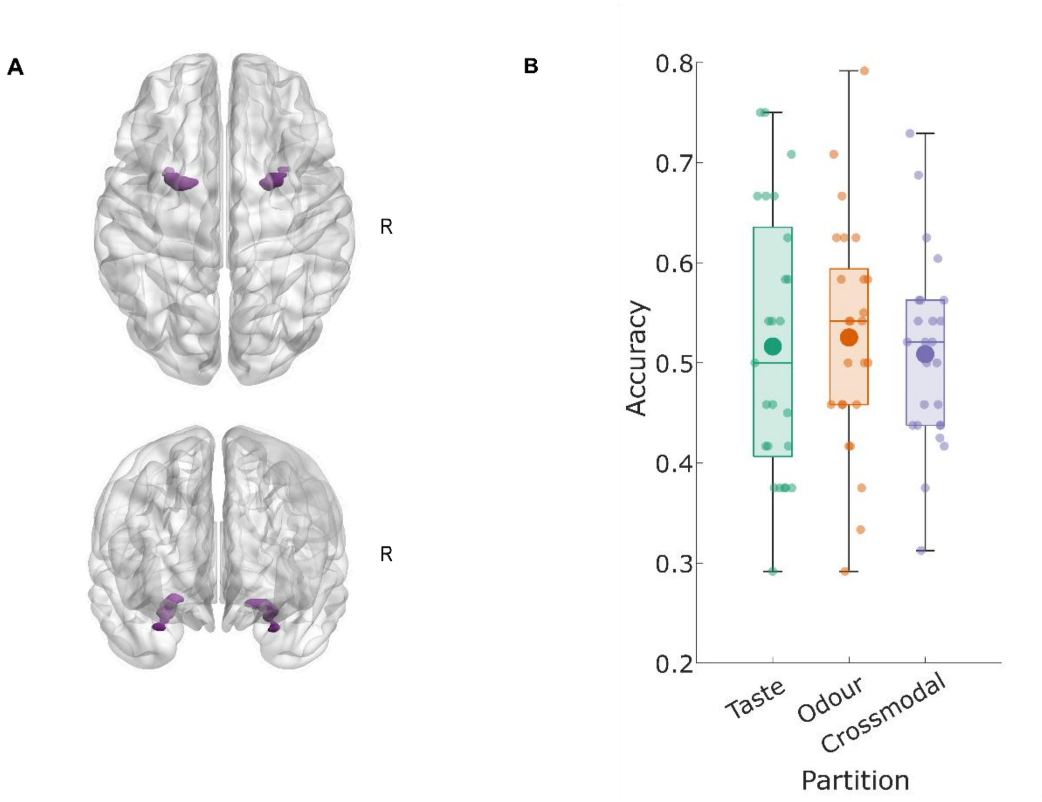

Supplementary Figure 6. ROI decoding in the piriform cortex. **A)** Axial (*top*) and coronal (*bottom*) views of the piriform ROI. **B)** Decoding accuracies in the piriform ROI. Decoding accuracy was not significantly above chance when distinguishing taste (p = .249) or odour (p = .138). Neither was crossmodal decoding significantly above chance (p = .281, all uncorrected one-tailed comparisons to permuted null distribution). N = 25 participants.

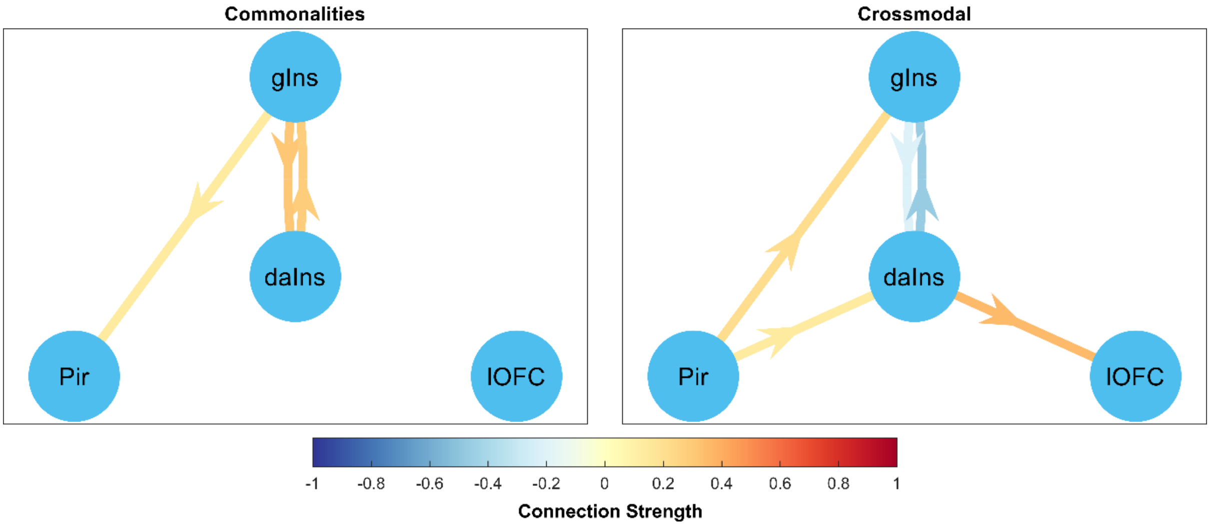

Supplementary Figure 7. Effective connectivity between the granular insula (gIns), dysgranular/agranular insula (daIns), piriform cortex (Pir) and the lateral orbitofrontal cortex (lOFC), with the daIns crossmodal accuracy scores as a second-level covariate. *Left (Commonalities).* Connectivity Bayesian parameter estimates in the DCM of the four ROIs common to all participants at the second-level Parametric Empirical Bayes (PEB), orthogonal to individual crossmodal decoding accuracy in the daIns. *Right (Crossmodal).* Connectivity Bayesian parameter estimates significant for the daIns crossmodal decoding accuracy covariate at the second-level PEB. Only significant connections thresholded at a posterior probability (Pp) > .95 are visualised. Arrow colours signify the parameter estimates of the connections (positive in red, negative in blue). N = 25 participants.

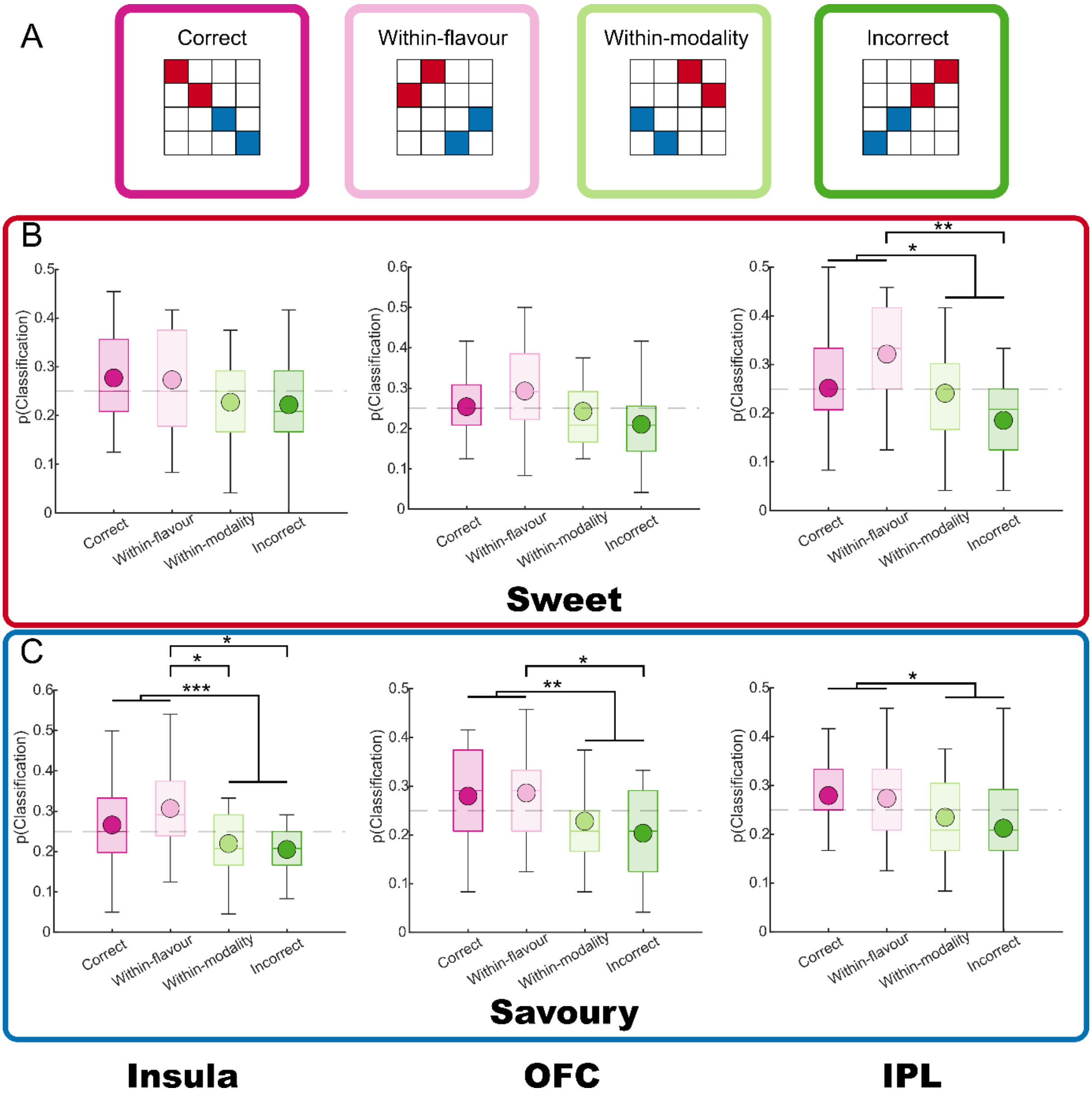

Supplementary Figure 8. Ancillary analyses of ROI confusion matrices. **A)** Graphical explanation of the various types of confusion within the confusion matrix, namely correct identification of stimuli, within-flavour confusion, within-modality confusion and incorrect classification, with sweet stimuli in red and savoury in blue. **B)** Breakdown of the confusion matrices in the three ROIs for the sweet stimuli. **C)** Breakdown of the confusion matrices in the three ROIs for the savoury stimuli. OFC – orbitofrontal cortex; IPL – inferior parietal lobule. Large dots signify means; boxes show the interquartile ranges (IQR); whiskers show 1.5 × IQR. Grey dashed lines signify theoretical chance accuracy. * $p < .05$, ** $p < .01$, *** $p < .001$, corrected with Tukey's Honest Significant Differences. N = 25 participants. Precise p-values are provided in the Source Data file.

Supplementary Table 1.

Linear Mixed Effects Model of Stimulus Identification Accuracy across Modality and Session

Outcome	Fixed Effect Structure	Estimate	Standard Error	*p*	Grouping Factor	Random Effect Structure
**Identification Accuracy**	Intercept	0.9383	0.036	7×10^{-64}	Subject ID	Intercept
	Session	0.0008	0.026	.976		Session
	Modality	0.0022	0.048	.963		Modality
	Session × Modality	0.0238	0.034	.486		Session × Modality

Supplementary Table 2.

Univariate unimodal BOLD activations against Artificial Saliva (ArtS)

Peak Structure	Peak *x*	Peak *y*	Peak *z*	Volume (mm^3)	*Z*	p_{FWE}
**Odour > ArtS**						
**Piriform Cortex**	-18	-2	-14	414	5.56	.0221
**Piriform Cortex**	26	0	-16	258	4.17	.0047 *SVC*
**Taste > ArtS**						
**Mid-Insula**	-36	-10	10	1019	5.16	.0054
**Mid-Insula**	36	-6	12	744	4.66	.0126
**Anterior Insula**	40	4	-8	234	4.46	.0096 *SVC*

SVC: small-volume correction on pre-defined coordinates (see **Methods**)

Supplementary Table 3.

Linear Mixed Effects Model of Classification Accuracy across Training and Testing sets

Outcome	Fixed Effect Structure	Estimate	Standard Error	*p*	Grouping Factor	Random Effect Structure
**Taste**						
**Accuracy**	Intercept	0.5712	0.029	3×10^{-34}	Subject ID	Intercept
	Testing Day	-0.0625	0.040	.121		Testing Day
	Training Day	-0.0490	0.038	.200		Training Day
	Testing Day × Training Day	0.1201	0.059	.044*		Testing Day × Training Day
**Odour**						
**Accuracy**	Intercept	0.5295	0.037	4×10^{-25}	Subject ID	Intercept
	Testing Day	0.0243	0.035	.492		Testing Day
	Training Day	0.0024	0.044	.956		Training Day
	Testing Day × Training Day	-0.0125	0.064	.846		Testing Day × Training Day
**Crossmodal**						
**Accuracy**	Intercept	0.5347	0. 025	1×10^{-34}	Subject ID	Intercept
	Testing Day	-0.0278	0.034	.410		Testing Day
	Training Day	0. 0094	0.035	.791		Training Day
	Testing Day × Training Day	-0. 0212	0.059	. 722		Testing Day × Training Day

* $p < .05$

Supplementary Table 4.

TFCE-corrected Whole-brain Searchlight Crossmodal MVPA Decoding

Peak Structure	Peak *x*	Peak *y*	Peak *z*	Volume (mm^3)	*Z*	P_{TFCE}
**Inf Parietal Lobule (A39rd)**	38	-74	31	24842	2.82	.0024
**Mid Frontal Gyrus (A9/46v)**	46	48	18	19774	2.23	.0211
**Precentral Gyrus (A6cvl)**	50	10	38	1929	1.86	.0314
**Orbital Gyrus (A11l)**	-16	27	20	738	1.85	.0320
**Fusiform Gyrus (A37lv)**	44	68	-16	1281	1.81	.0350
**Post Sup Temporal Sulcus (rpSTS)**	54	-36	-10	1960	1.79	.0364
**Insular Gyrus (dla)**	36	28	8	124	1.74	.0410
**Inf Parietal Lobule (A40v)**	64	-22	-36	318	1.74	.0413
**Orbital Gyrus (A11l)**	14	44	24	212	1.68	.0464

Brackets enclose the cytoarchitectonic descriptions of the peak from the Brainnetome Atlas.

Supplementary Table 5.

TFCE-corrected Whole-brain Searchlight Taste MVPA Decoding

Peak Structure	Peak *x*	Peak *y*	Peak *z*	Volume (mm^3)	*Z*	P_{TFCE}
**Postcentral Gyrus (A2)**	58	-12	36	169179	3.06	.0011
**Occipital Gyrus (V5/MT)**	46	-72	-2	53935	2.66	.0039
**Postcentral Gyrus (A2)**	-40	-32	56	2323	2.03	.0211
**Cingulate Gyrus (A24rv)**	4	-4	28	236	1.90	.0286
**Striatum (dCa)**	20	10	14	330	1.87	.0304
**Mid Frontal Gyrus (A8vl)**	-34	28	50	539	1.81	.0348
**Mid Frontal Gyrus (A6vl)**	38	10	62	1101	1.77	.0380
**Sup Frontal Gyrus (A8dl)**	22	26	44	234	1.73	.0416
**Sup Frontal Gyrus (A6dl)**	-22	-4	60	393	1.71	.0436
**Inf Parietal Lobule (A39rd)**	42	-62	48	223	1.69	.0455
**Mid Temporal Gyrus**	54	4	-14	389	1.68	.0466

Brackets enclose the cytoarchitectonic descriptions of the peak from the Brainnetome Atlas.
