# Supplementary material for: Tastes and retronasal odours evoke a shared flavour-specific neural code in the human insula
Source: Nat Commun. 2025 Sep 12;16:8252. doi: 10.1038/s41467-025-63803-6 (PMC12432251; doi:10.1038/s41467-025-63803-6)
Supplement: Supplementary file 2 — Reporting Summary [file 41467_2025_63803_MOESM2_ESM.pdf]

nature portfolio

Corresponding author(s): *Double-anonymous peer review submissions: write DAPR and your manuscript number here instead of author names.*

Last updated by author(s): *YYYY-MM-DD*

Reporting Summary

Nature Portfolio wishes to improve the reproducibility of the work that we publish. This form provides structure for consistency and transparency in reporting. For further information on Nature Portfolio policies, see our Editorial Policies and the Editorial Policy Checklist.

Statistics

For all statistical analyses, confirm that the following items are present in the figure legend, table legend, main text, or Methods section.

n/a	Confirmed	
☐	☒	The exact sample size (*n*) for each experimental group/condition, given as a discrete number and unit of measurement
☐	☒	A statement on whether measurements were taken from distinct samples or whether the same sample was measured repeatedly
☐	☒	The statistical test(s) used AND whether they are one- or two-sided<br>*Only common tests should be described solely by name; describe more complex techniques in the Methods section.*
☐	☒	A description of all covariates tested
☐	☒	A description of any assumptions or corrections, such as tests of normality and adjustment for multiple comparisons
☐	☒	A full description of the statistical parameters including central tendency (e.g. means) or other basic estimates (e.g. regression coefficient) AND variation (e.g. standard deviation) or associated estimates of uncertainty (e.g. confidence intervals)
☐	☒	For null hypothesis testing, the test statistic (e.g. *F*, *t*, *r*) with confidence intervals, effect sizes, degrees of freedom and *P* value noted<br>*Give P values as exact values whenever suitable.*
☐	☒	For Bayesian analysis, information on the choice of priors and Markov chain Monte Carlo settings
☐	☒	For hierarchical and complex designs, identification of the appropriate level for tests and full reporting of outcomes
☐	☒	Estimates of effect sizes (e.g. Cohen's *d*, Pearson's *r*), indicating how they were calculated

Our web collection on statistics for biologists contains articles on many of the points above.

Software and code

Policy information about availability of computer code

Data collection	Data collection used custom Psychtoolbox-3 code implemented in MATLAB 2021b (Mathworks).
Data analysis	Images were preprocessed using SPM12 (Wellcome Department of Imaging Neuroscience, Institute of Neurology, London, UK) implemented in MATLAB 2021b (Mathworks). First-level GLMs were constructed using SPM12. Second-level univariate analyses were conducted using SPM12. Decoding and whole-brain TFCE of accuracy maps was conducted using the CosMoMVPA toolbox. Permutation testing of ROI decoding accuracy was performed using custom scripts available on https://osf.io/2kryv/

For manuscripts utilizing custom algorithms or software that are central to the research but not yet described in published literature, software must be made available to editors and reviewers. We strongly encourage code deposition in a community repository (e.g. GitHub). See the Nature Portfolio guidelines for submitting code & software for further information.

Data

Policy information about availability of data

All manuscripts must include a data availability statement. This statement should provide the following information, where applicable:

- Accession codes, unique identifiers, or web links for publicly available datasets
- A description of any restrictions on data availability
- For clinical datasets or third party data, please ensure that the statement adheres to our policy

The first-level betas generated in this study have been deposited in the Zenodo database [DOI: 10.5281/zenodo.16875496; https://zenodo.org/records/16875496]. Access can be obtained by filling out a guest access request. Unthresholded group-level data is available on https://neurovault.org/collections/OOBZESPX/. Source

Data are provided with this paper.

Research involving human participants, their data, or biological material

Policy information about studies with human participants or human data. See also policy information about sex, gender (identity/presentation), and sexual orientation and race, ethnicity and racism.

Reporting on sex and gender	Male and female volunteers participated in the study. Of the 25 included, 11 were male and 14 were female, as determined by self-report. As we had no a priori hypotheses involving either sex or gender, no sex- or gender-based analyses were performed.
Reporting on race, ethnicity, or other socially relevant groupings	We did not use race/ethnicity or similar socially constructed variables. Neither did we collect data on participants' ethnicity. We collected data on the number of years of education participants had completed at the time of the experiment.
Population characteristics	The mean age of the sample was 27.8 years (SD = 6.0 years), and the mean BMI was 22.8 kg/m2 (SD = 2.8 kg/m2). To be included, participants had to be between 18 and 45 years old, speak fluent English, have a normal sense of smell (tested using the Sniffin' Sticks Identification task [cut-off score of 12]) and a normal sense of taste (tested using tastant sprays in the mouth), have normal or corrected-to-normal vision, not be pregnant, display no cold/flu symptoms and have no known eating disorder.
Recruitment	Participants were recruited by flyers placed all around the Stockholm area. Our recruitment was intended to reach a representative sample of the population, without explicit biases imposed by the researchers. However, the geographical context in which the study was conducted, and the format of the research itself present risks for self-selection bias which need to be acknowledged. First, the city of Stockholm's strong educational infrastructure and knowledge-based job market, combined with our strategy of advertising primarily around university campuses and central areas of the city likely biased our sample towards a population with above-average educational attainment and economic power within national and international comparison. Second, the fact that our study required participants to attend three prescheduled experimental sessions required a level of flexibility from participants that likely represents a burden for those with a high load of professional or caretaking responsibility, who are therefore likely to not apply to be included. Finally, the MRI environment places restrictions on recruitment of people with preexisting health conditions such as back pain, or mental health problems such as claustrophobia, biasing towards a sample with above average physical and mental health.
Ethics oversight	All procedures were in accordance with the Declaration of Helsinki and approved by the local ethics committee (Regionala etikprövningsnämnden i Stockholm, Dnr 2021-05138).

Note that full information on the approval of the study protocol must also be provided in the manuscript.

Field-specific reporting

Please select the one below that is the best fit for your research. If you are not sure, read the appropriate sections before making your selection.

☐ Life sciences ☒ Behavioural & social sciences ☐ Ecological, evolutionary & environmental sciences

For a reference copy of the document with all sections, see nature.com/documents/nr-reporting-summary-flat.pdf

Behavioural & social sciences study design

All studies must disclose on these points even when the disclosure is negative.

Study description	Quantitative cross-sectional
Research sample	The sample consisted of Stockholm residents. Out of the 25 participants, 11 were male and 14 were female based on self-report. The mean age of the sample was 27.8 years (SD = 6.0 years), and the mean BMI was 22.8 kg/m2 (SD = 2.8 kg/m2). To be included, participants had to be between 18 and 45 years old, speak fluent English, have a normal sense of smell (tested using the Sniffin' Sticks Identification task [cut-off score of 12]) and a normal sense of taste (tested using tastant sprays in the mouth), have normal or corrected-to-normal vision, not be pregnant, display no cold/flu symptoms and have no known eating disorder. This sample is potentially not fully representative, due to the gender imbalance and limitations on smoking and normosmia.
Sampling strategy	Recruitment was conducted via flyers posted in the general Stockholm area. We aimed to recruit 30 participants, based on both funding constraints (funding would only cover 30 participants for three visits) and this sample size being comparable to previous studies in the field (e.g. Suzuki et al., 2017; Avery et al., 2020; Khorisantono et al., 2023).
Data collection	Behavioural session: Prior to the behavioural session, participants were randomly assigned two abstract visual cues (letters from the Phoenician alphabet), one for the savoury flavour and another for the sweet flavour. In each trial of the rating task, they participant was presented 1 ml of a stimulus in taste cups and was asked to choose which visual cue it corresponded to, such that participants would learn the cue associated with each flavour. This cue association allowed us to examine if participants were able to distinguish the flavours without being primed by words such as 'sweet' or 'savoury'. They subsequently rated it for pleasantness and intensity. Parts of the trial that required participant input ('Identify', 'Pleasantness' and 'Intensity') lasted for a maximum of 5 seconds but ended after the participant entered the input. Therefore, each trial ranged between 10 s to 25 s. Participants completed up to 60 trials, although from trial 40 onwards the experiment ended once their cumulative accuracy rate was above 85%, thereby ensuring that the participants could sufficiently distinguish between the two flavour stimuli. In the behavioural sessions, all tasks were performed on a computer using a keyboard extension. In the first part, participants completed the task sitting up. In the second,

participants were lying down on a massage bench with a mouthpiece connected to a gustometer that delivered the stimuli orally. They performed the task using a button box and visual stimuli were presented on an iPad.

MRI sessions: Participants were instructed to attend two structural and functional MR sessions, scheduled such that the final MR session was within 10 days of the behavioural session. During the MRI sessions, participants orally received unimodal stimuli – that is, only sweet taste (SweT), savoury taste (SavT), 'sweet' odour (SweO) or 'savoury' odour (SavO) during the mini-block, as opposed to a flavour combination, in addition to artificial saliva. Each participant had bespoke SweO and SavO stimuli derived from the flavour combinations used in their behavioural laboratory session. Participants were presented with 0.5 ml of the unimodal stimuli or artificial saliva and asked to swallow. This sequence was repeated four times with the same stimulus to form a mini-block (for a total of 2 ml over 16 seconds) and followed by a rinse block consisting of 1 ml of artificial saliva before moving to the inter-trial interval. Between mini-blocks, participants were presented with a grey fixation cross for 8-12 s. Participants were instructed to swallow only when the swallow cue appeared on the screen. The stimulus order was pseudo-randomised such that every stimulus (including artificial saliva) has 3 repetitions per run and that stimuli were not repeated consecutively more than once, and each run consisted of 15 mini-blocks. In all cases, participants were blind to the hypotheses of the experiment. Experimenters were aware of the hypotheses of the experiment, but all stimulus presentations were randomised by a computer and experimenters had no control over which stimulus was presented. Only the experimenters and participants were present for all sessions.

Timing

The first participant attended their behavioural session on 3 July 2023, and the last participant completed their scanning sessions on 23 December 2023.

Data exclusions

Out of the 28 participants scanned, 3 had to be excluded for: excessive signal dropout (1), excessive movement during scanning (1) and nausea (1). Within each participant, runs with more than 10% of their frames censored due to excessive motion were excluded from analysis.

Non-participation

We recruited 40 participants with an expected 25% dropout rate, but only 28 of those recruited completed the scanning sessions. Of those who did not continue to complete the scanning sessions, 4 found the stimuli disgusting; 1 did not pass the Sniffin' Sticks identification test; 1 did not pass the tastant spray test; 2 dropped out prior to scanning due to claustrophobia; 1 did not attend the sessions with no information; 1 was a smoker; 2 performed below 75% in the behavioural task.

Randomization

Participants were not allocated into separate groups.

Reporting for specific materials, systems and methods

We require information from authors about some types of materials, experimental systems and methods used in many studies. Here, indicate whether each material, system or method listed is relevant to your study. If you are not sure if a list item applies to your research, read the appropriate section before selecting a response.

Materials & experimental systems

n/a	Involved in the study
x	Antibodies
x	Eukaryotic cell lines
x	Palaeontology and archaeology
x	Animals and other organisms
x	Clinical data
x	Dual use research of concern
x	Plants

Methods

n/a	Involved in the study
x	ChIP-seq
x	Flow cytometry
	x MRI-based neuroimaging

Plants

Seed stocks

Report on the source of all seed stocks or other plant material used. If applicable, state the seed stock centre and catalogue number. If plant specimens were collected from the field, describe the collection location, date and sampling procedures.

Novel plant genotypes

Describe the methods by which all novel plant genotypes were produced. This includes those generated by transgenic approaches, gene editing, chemical/radiation-based mutagenesis and hybridization. For transgenic lines, describe the transformation method, the number of independent lines analyzed and the generation upon which experiments were performed. For gene-edited lines, describe the editor used, the endogenous sequence targeted for editing, the targeting guide RNA sequence (if applicable) and how the editor was applied.

Authentication

Describe any authentication procedures for each seed stock used or novel genotype generated. Describe any experiments used to assess the effect of a mutation and, where applicable, how potential secondary effects (e.g. second site T-DNA insertions, mosiacism, off-target gene editing) were examined.

Magnetic resonance imaging

Experimental design

Design type

We collected task-based fMRI data using a mini-block design, where each event (stimulus delivery) was conducted in a mini-block of 16 s.

Design specifications

Participants were instructed to attend two structural and functional MR sessions, scheduled such that the final MR session was within 10 days of the behavioural session. During the MRI sessions, participants orally received unimodal stimuli – that is, only sweet taste (SweT), savoury taste (SavT), 'sweet' odour (SweO) or 'savoury' odour (SavO) during the mini-block, as opposed to a flavour combination, in addition to artificial saliva. Each participant had bespoke SweO and SavO stimuli derived from the flavour combinations used in their behavioural laboratory session. Participants completed six functional runs per session. Participants were presented with 0.5 ml of the unimodal stimuli or artificial saliva and asked to swallow. This sequence was repeated four times with the same stimulus to form a mini-block (for a total of 2 ml over 16 seconds) and followed by a rinse block consisting of 1 ml of artificial saliva before moving to the inter-trial interval. Between mini-blocks, participants were presented with a grey fixation cross for 8-12 s. Participants were instructed to swallow only when the swallow cue appeared on the screen. The stimulus order was pseudo-randomised such that every stimulus (including artificial saliva) has 3 repetitions per run and that stimuli were not repeated consecutively more than once, and each run consisted of 15 mini-blocks.

Behavioral performance measures

We elected a passive tasting paradigm, so participants did not report anything via button presses. However, each MRI session began with a shortened version of the behavioural task from the laboratory session (24 trials) where they performed the identification and rating tasks in the MR scanner bore, prior to any scanning, using the same visual cues that they were assigned in the laboratory session. Participants were briefed that the flavours they would receive might vary slightly from the ones in the laboratory session, such as in terms of intensity. Only participants who scored over 75% accuracy could continue. All participants achieved this score.

Acquisition

Imaging type(s)

Functional; structural

Field strength

3.0 T

Sequence & imaging parameters

Each run consisted of 228 T2*-weighted BOLD gradient multi-echo echoplanar images (EPI) using a Siemens 3 T Prisma scanner running the syngo MR E11 system equipped with a 64-channel head coil. Fifty-two interleaved axial slices of 1.7 mm thickness were collected with the following parameters: in-plane voxel size = 2mm × 2 mm; slice thickness = 1.7 mm (distance factor of 15%); echo time (TE) = 42.0 ms; repetition time (TR) = 2000 ms; flip angle (FA) = 90 deg; field of view (FOV) = 208 mm × 208 mm.

Prior to the functional scans, a gradient echo image with two echoes was extracted to generate a field map with the following parameters: 2 mm isotropic voxel; TE1 = 4.92 ms; TE2 = 7.38 ms; FA = 60 deg; TR = 565 ms; FOV = 208 mm × 208 mm; phase encoding direction: R → L axial.

Furthermore, to enable normalisation to a standardised space, a high-resolution multi-echo T1-weighted MPRAGE structural image was acquired with the following parameters: 1 mm isotropic resolution sagittal slices; TE1 = 1.69 ms; TE2 = 3.55 ms; TE3 = 5.41 ms; TE4 = 7.27 ms; FA = 7.0 deg; acquisition time = 2530 s; FOV = 256 mm × 256 mm. Scanner-side extraction of the root-mean-square (RMS) of the multiple echoes resulted in a high-quality image that was used in subsequent steps.

Area of acquisition

We aimed to collect whole-brain data. For brains that exceeded the field of view, partial brain coverage was used, which included the frontal lobe, temporal lobe and occipital lobe.

Diffusion MRI ☐ Used ☒ Not used

Preprocessing

Preprocessing software

Images were preprocessed using SPM12 (Wellcome Department of Imaging Neuroscience, Institute of Neurology, London, UK) implemented in MATLAB 2021b (Mathworks). Firstly, a field map for each MRI session was calculated using the gradient echo images, specifically the phase images and the magnitude image, using the in-built SPM12 Field Map Toolbox. Random EPI images from the session were then loaded and unwarped to visually check the extent of distortion correction. The structural images from both MRI sessions were coregistered and a mean image was extracted in order to further improve spatial resolution. This mean structural image was then segmented into tissue probability maps (TPMs), and a skull-stripped brain consisting of the grey matter, white matter and cerebrospinal fluid was calculated (with a combined TPM cutoff of 0.8).

After slice-time interpolation to the 0.99-second slice, functional images from each run were realigned to the first image and unwarped using the previously calculated field map for the pertinent session. They were then coregistered to the skullstripped brain and . The resultant normalised images were then smoothed with a 6 mm full-width-half-maximum (FWHM) isotropic Gaussian kernel. Finally, both the smoothed and unsmoothed normalised images from each run were detrended using Linear Model of Global Signal detrending to remove global effects from the time series on top of the pre-registered preprocessing pipeline. These detrended images were used in further analyses.

Normalization

Data were non-linearly normalised to MNI space using the deformation field obtained from the segmentation phase, using the spm_normalise function.

Normalization template

MNI305 (based on the SPM tissue probability maps)

Noise and artifact removal

Data were detrended by modelling the global signal such that global effects were removed from the time series. Further artifact signals were removed by using the six motion regressors (x, y, z, pitch, roll and yaw) and spike regressors for censored frames in the main general linear model.

Volume censoring

Volumes were censored if they exceeded 1 mm framewise displacement. Censoring was performed by adding a spike regressor for each censored volume in the GLM. Runs that had more than 10% of its volumes censored were excluded from analysis.

Statistical modeling & inference

Model type and settings

Mass-univariate GLM analyses: First-level general linear models (GLMs) were conducted within each participant using the smoothed and normalised individual BOLD data in SPM12. Within each individual subject, we modelled the following regressors of interest (using a boxcar function with a duration of 16 s):
• All tastant presentations (both sweet and savoury – Condition 1)
• All odorant presentations (both 'sweet' and 'savoury' – Condition 2)
• All ArtS presentations as the explicit baseline condition (Condition 3)
We also modelled the rinse block for a period of 4 s as a regressor of no interest. The boxcar regressors were convolved with the canonical haemodynamic response basis function (HRF). Furthermore, confound regressors included the six rigid-body motion parameters estimated from the realignment procedure, in addition to spike regressors to censor frames with framewise displacement greater than 1 mm (as calculated from the motion parameters). Runs with more than 10% of their frames censored were excluded from the analysis. A grey-matter explicit mask was used (SPM grey matter TPM thresholded at 0.2) to limit the analysis to only voxels containing grey matter. We also specified the model to include a high-pass filter (HPF) of 128 s to remove slow signal drift and an autocorrelation function of AR(1).
Within each subject, a contrast of interest (Condition 1 – Condition 3 in the case of tastants and Condition 2 – Condition 3 in the case of odorants) was calculated. The group-level analysis using a one-sample t-test on the globally pooled contrast of interest using an unweighted summary statistics approach. Analyses were conducted using a whole-brain grey-matter mask. Cluster-wise significance was conducted using a threshold of PFWE < .05, a cluster-cutting threshold of k > 15 and voxel-wise threshold of uncorrected P < .001. Small-volume corrections (SVCs) were performed from pre-registered peaks, limiting them to a 10 mm radius spherical Region of Interest (ROI). ROIs for small-volume corrections and the analysis steps were pre-registered, although we applied a stricter cluster-cutting threshold than pre-registered.

Multivariate pattern analysis: Multivariate Pattern Analysis (MVPA) was conducted using the CoSMoMVPA toolbox implemented in MATLAB 2021b. For ROI analyses, the ROIs were formed from functional clusters of the contrast of tastants against ArtS in the mass-univariate GLM analysis to isolate regions that were responsive to taste. In order to avoid bias by using the same participant for ROI generation and analysis, ROIs for each subject were generated from the data of the remaining subjects (leave-one-subject-out cross-validation), using a more lenient voxel-wise threshold of uncorrected P < .01 and a cluster-cutting threshold of k > 150. Post-hoc ROI MVPA used subregions of the insula as parcellated by Fan et al. (2016).
Prior to any MVPA, first-level GLM analyses were conducted on unsmoothed normalised functional data in order to preserve voxel-level differences in activation. Within each subject, we used the following regressors of interest (using a boxcar function with a duration of 16 s):
• Sweet taste presentation (SweT)
• Savoury taste presentation (SavT)
• 'Sweet' odour presentation (SweO)
• 'Savoury' odour presentation (SavO)
• Artificial saliva explicit baseline presentation (ArtS)
In addition, rinse periods were modelled as regressors of no interest for a period of 4 s. Regressors were convolved with the canonical HRF basis and confound regressors were the same as the univariate analyses. Similar to the univariate analyses, we also included an HPF of 128 s and applied a whole-brain grey-matter mask.
MVPA was conducted on the resultant betas of the above model. As pre-registered, we trained a support vector machine (SVM) decoder on the beta weights for each voxel obtained from the first-level GLM. We employed a leave-one-run-out cross-validation partition: in each decoding step, the decoder is trained on all runs bar one, and its performance is subsequently tested on the left-out run. For crossmodal decoding analyses, we subtracted the training data mean from both the training and testing data to remove mass univariate differences in activation between different modalities. Whole-brain crossmodal searchlight MVPA used the unsmoothed detrended functional scans in MNI space. At each spherical searchlight with a radius of 4 voxels (8 mm), we applied the same partitioning and mean-centring strategy as the ROI analysis to train and test the decoder. Only grey-matter voxels were used to create the searchlights. We then mapped the average accuracy of the decoder onto the centre voxel of the searchlight before moving onto the next searchlight. We subtracted the theoretical chance level from the accuracy maps before then smoothing them using a 6mm FWHM isotropic Gaussian kernel for group-level analysis.

Effect(s) tested

Participants were orally presented sweet taste, savoury taste, sweet odour, savoury odour or artifical saliva in each mini-block. For univariate analyses on cortical responses to tastants, the effects examined were tastants vs artificial saliva. For univariate analyses on cortical responses to odorants, the effects examined were odorants vs artificial saliva.
For ROI multivariate analyses, a decoder was trained to differentiate the tastants. Another was trained to differentiate the odorants. Finally, one was trained on the tastant data and tested on odorants and vice versa.

Specify type of analysis: ☐ Whole brain ☐ ROI-based ☒ Both

Anatomical location(s)

The main multivariate analysis used taste-responsive clusters in the bilateral insula. This method used leave-one-subject-out cross-validation, where the ROI cluster for each subject was independently generated using the tastant map of all subjects bar the tested subject.

Decoding using the parcellated ROIs (to differentiate between the granular insula and the agranular and dysgranular portions) was performed using the parcellations of the Brainnetome atlas (Fan et al. 2016).

Statistic type for inference

(See Eklund et al. 2016)

For univariate analyses, cluster-wise significance testing was conducted using a threshold of PFWE < .05, a cluster-cutting threshold of k > 15 and voxel-wise threshold of uncorrected P < .001. For prevalence testing of accuracy maps from multivariate pattern analysis, we used threshold-free cluster enhancement (TFCE) which resulted in a z-map. Clusters greater than 15 contiguous voxels oof z greater than 1.65 (for one-tailed significance at alpha = .05) were considered significant.

Correction	For univariate analyses, familywise-error correction was applied using random field theory. For prevalence testing of accuracy maps from searchlight multivariate pattern analysis, we performed prevalence testing on the group data using 10000 Monte-Carlo simulations and threshold-free cluster enhancement to account for multiple comparisons (Smith & Nichols, 2017) implemented in CoSMoMVPA.

Models & analysis

n/a	Involved in the study
☐	☒ Functional and/or effective connectivity
☒	☐ Graph analysis
☐	☒ Multivariate modeling or predictive analysis

Functional and/or effective connectivity	We used Dynamic Causal Modelling (DCM) to characterise associations between crossmodal decoding accuracy in the dysgranular/agranular insula (daIns) and directed information flow in an anatomically defined network of ROIs, namely the granular insula (gIns), daIns, piriform cortex (Pir) and lOFC. A fully connected DCM was constructed with driving inputs to the gIns for taste stimuli, Pir for the odour stimuli and both gIns and Pir for the ArtS condition. In addition, the DCM had a full intrinsic, steady-state and bidirectional connectivity matrix (A matrix) between all four regions. We did not specify modulatory effects on the connections between regions. After Volume of Interest (VOI) extraction from each specified region in each participant (concatenating the design matrix across runs with run-specific intercepts), we estimated the full DCM for each participant. We then used second-level parametric empirical Bayes approach (PEB) to prune the model to a set of parameters that best explain the data73, with a constant intercept and each participant's daIns crossmodal accuracy in the design matrix. This results in a Bayesian hierarchical model with estimates for each connection specified in the A matrix, as well as the posterior probabilities derived by comparing the evidence for all models in which the specific connection was switched on versus all models where it was switched off74. Parameters were estimated for the commonalities (average connectivity across participants, orthogonal to crossmodal decoding accuracy in daIns) as well as the connections associated with crossmodal decoding accuracy in daIns. We displayed connections thresholded at a posterior probability (Pp) > .95 for the corresponding parameter.
Multivariate modeling and predictive analysis	We generated a GLM with quality-specific regressors for odours and tastants, i.e., one regressor each for sweet taste (SweT), savoury taste (SavT), sweet odour (SweO), savoury odour (SavO) and artificial saliva (ArtS) and trained a support vector machine (SVM) on the resultant betas from all runs but one (leave-one-run-out cross-validation). For crossmodal decoding, we trained a classifier on taste quality and tested it on odour quality and vice versa. Classifier performance was quantified based on is accuracy.
